# Mechanical resistance of zirconium implant abutments: A review of the literature

**DOI:** 10.4317/medoral.17462

**Published:** 2011-12-06

**Authors:** Rocio Velázquez-Cayón, Cristina Vaquero-Aguilar, Daniel Torres-Lagares, Manuel Jiménez-Melendo, José L. Gutiérrez-Pérez

**Affiliations:** 1 Research. Faculty of Dentistry. University of Seville; 2 Research. Department of Physics. University of Seville; 3 Professor. Faculty of Dentistry. University of Seville; 4 Professor. Department of Physics. University of Seville; 5 Head. University Hospital Virgen del Rocío, Oral and Maxillofacial Surgery Unit, Seville

## Abstract

The increase of aesthetic demands, together with the successful outcome of current implants, has renewed interest in the search for new materials with enough mechanical properties and better aesthetic qualities than the materials customarily used in implanto-prosthetic rehabilitation. Among these materials, zirconium has been used in different types of implants, including prosthetic abutments. The aim of the present review is to analyse current scientific evidence supporting the use of this material for the above mentioned purposes. We carried out the review of the literature published in the last ten years (2000 through 2010) of in vitro trials of dynamic and static loading of zirconium abutments found in the databases of Medline and Cochrane using the key words zirconium abutment, fracture resistance, fracture strength, cyclic loading. Although we have found a wide variability of values among the different studies, abutments show favourable clinical behaviour for the rehabilitation of single implants in the anterior area. Such variability may be explained by the difficulty to simulate daily mastication under in vitro conditions. The clinical evidence, as found in our study, does not recommend the use of implanto-prosthetic zirconium abutments in the molar area.

** Key words:** Zirconium abutment, zirconium implant abutment, zirconia abutment, fracture resistance, fracture strength, cyclic loading.

## Introduction

Implantology has been one of the greatest odontological achievements in the last century. Once the stage when osteointegration was the only key to success has been overcome, the present challenge is to ensure both the functionality and the aesthetic value of the implant-supported prosthesis. 

The advantages of using ceramic components in implantoprosthetic rehabilitation can be summarized in the possibility to individualize the components, to take full advantage of their light transmission properties, excellent aesthetic qualities with colours resembling those of teeth and their great compatibility with preimplant soft tissues. Nevertheless, there are also disadvantages such as their lesser resistance to fatigue (1). 

Nowadays most companies in the sector offer ceramic abutments which may be prefabricated or manufactured and can also be prepared in a dental laboratory either by a technician following the traditional method or with the assistance of computer-based designs. The materials chosen for that purpose are: highly pure alumina (Al2O3), TZP ceramics (stabilized tetragonal zirconia) or PSZ ceramics (partially stabilized zirconia). TZP ceramics show superior properties to alumina due to such microstructural differences as density, size of particles (smaller in the case of TZP) and their polymorphic mechanism against flaw propagation. The main reason for such superiority is the yttrium stabilization of the zirconia structure (2).

The production of large components of pure zirconium is not possible. The extensive volume of the transformation between the different stages of the zirconium may be an advantage due to the addition of cubic stabilizing oxides (the most common are yttrium oxide, magnesia and calcium oxide). These oxides can stabilize a relatively weak cubic structure below room temperature. On the other hand, if we add enough amount of stabilizing oxide, and the material is correctly processed, zirconium particles may be stored in a metastable tetragonal form at room temperature. All these materials are known as partially stabilized zirconium ceramics (PSZ). The transformation from the tetragonal to the monoclinic structure may serve to improve the mechanical resistance and fracture tenacity of PSZ ceramics. Used under stress in the area of a flaw, metastable tetragonal particles pass to a stable monoclinic structure, change which is known as martensitic transformation. The extension of the resulting volume is distributed around these particles close to the flaw thus compressing it and delaying its propagation until the stress is increased. This physical phenomenon ensures the reliability of this material for the production of implant-supported abutments (3). 

Zirconium oxide, or zirconia, shows similar mechanical properties to metals and its colour resembles that of teeth. Partially yttrium-stabilized zirconia shows the best properties for the purposes under discussion. Zirconia was first used with medical pur-poses in 1969. Helmer and Driskell introduced it as a new material for orthopaedic uses in the Symposium on Use of Ceramics as Surgical Implants. They presented it as a substitute for titanium or alumnia prostheses in hip replacement. The first zirconia abutment, Zirabut® (Wolhwend Innovative, Zurich, Switzerland) was produced in 1997, although many more were added later (4).

The aim of the present review is to confirm scientific evidence in favour of the resistance of partially yttrium-stabilized zirconia abutments, commonly known simply as zirconia abutments, using dynamic and static loading trials. 

## Material and Methods

We carried out the review of the literature published during the last ten years (2000-2010) of in vitro trials of dynamic and static loading of zirconium abutments. In order to do so, we used the databases of Medline (Pubmed) and Cochrane. The key words used in the search were: zirconium abutment, zirconium implant abutment, zirconia abutment, fracture resistance, fracture strength, cyclic loading. First of all, we searched zirconium abutment OR zirconium implant abutment OR zirconia abutment and then we looked for fracture resistance OR fracture strength OR cyclic loading. Finally, we combined the keywords of both searches with the operator AND. 

In order to complete the search, we carried out a manual examination in the following publications of the articles available on the subject published in the last ten years: Journal of Prothetic Dentistry, Journal of Oral Rehabilitation, Quintessence International, Journal of Prosthodontics, International Journal of Oral Maxillofacial Implants, International Journal of Prosthodontics.

We examined all the studies including in vitro fracture strength at a constant speed and dynamic loading trials of zirconia abutments used as implantoprosthetic abutment (with or without comparison with other type of material). We considered both studies in which the implantoprosthetic system was completely rehabilitated and those in which it was not. 

## Results

We found 9 articles whose main characteristics are shown in ( Table 1).

In one of the trials on zirconia abutments (5) (Wohlwend Innovative, Switzerland) a computer controlled universal testing device was used and the load was applied at 30º from the axial axis of the implant-supported system. The implant-abutment system was torqued to 32 N cm. The crown failed in 40% of the cases, the fixing screw in 30% of cases and in the remaining 30% the abutment itself failed. If we leave aside the abutments in which the fixing screw failed, mean fracture-load value was 788.1 N with an interval of 619.5- 1365.6 N. If we consider all the abutments, mean value was 737.6 N. 

Likewise, Butz and cols. (6) in their analysis of 16 abutments Zireal® (Biomet 3i Palm Beach Garden, Florida, US) fixed with gold screws (GoldTite®, Biomet 31 Palm Beach Garden, Florida, US) to 32 N cm and repaired with a Dentitan® crown (Krupp, Essen, Germany), obtained fracture load values within the range 240N-450N, with a mean value of 295 N; 25% of the implants failed as a consequence of abutment fracture, 13% due to fixing screw fracture and the remaining due to the detachment of the crown from the abutment. The same authors observed a fracture load between 180 N and 460 N with a mean value of 325 N, in the case of titanium abutments (GingiHue®, Biomet 3i, Palm Beach Garden, Florida, US) repaired with the same type of crown. 

Aramouni and cols. (7) in a recent study on ZiReal® abutments (Biomet 3i, Palm Beach Garden, Florida, US) with porcelain crowns tried at a constant speed of 1mm/min found that the crown failed in 80% of the cases. Such studies show that crown detachment is one of the most common causes of endosseous implant failure due to translation and rotation movements involved in the abutment-crown interface and the presence of the corresponding adhesive. Thus, applying successive coats of porcelain to cover the abutment seems an effective alternative to solve the problem. 

Gehrke and cols. (8) in a report on naked zirconia abutments Cercon® Dentsply/Friadent (Mannheim, Germany) of 4.5 mm in diameter and 18mm in length found a survival rate of up to 106 cycles, 1 Hz at loads of 100 to 450 N. Fracture resistance of these cycled abutments decreased by 60% (up to 270 N) in comparison to non-fatigued abutments. The authors do not explain the origin of the failure.

Adatia and cols. (9) published in 2009 an article on Astra Tech zirconia abutments (Y-TZP®, Sweden) made with different axial reductions (0.5 and 1mm). The authors observed a mean fracture resistance of 429 N±140; 576±120 for the reduction group of 0.5mm and 547±139 in the 1mm group. They concluded that axial reduction does not affect fracture resistance of zirconia abutments in a significant way.

Kerstein and Radke (10) compared two types of zirconia abutments: 29 Procera AllZirkon® abutments (Nobel Biocare, Sweden) and 29 AAZ® abutments (Atlantis®, AstraTech,Sweden) joined to regular platform titanium implants and torqued at 35N cm. The whole piece was applied increasing loads (beginning at 0N) until its fracture, with a 40º axis in relation to the axial plan. With a sweeping electron microscope, they analysed the origin of the fracture and its trajectory. Mean fracture force for AAZ® abutments was 831 N and 740 N for AllZirkon® samples.

In both instances, the fracture was due to small defects in their surface resulting from the fabrication process. AAZ® abutments had statistically lower probability of fracture at loads which resembled human mastication. However, both types of abutments failed at loads heavier than those occurring in daily mastication. 

Kim and cols. (11) studied an alternative to zirconia abutments obtained through CAD/CAM technique. It is a metal-ceramic abutment containing a biocompatible metal alloy followed by an IPS e.max Press® injection. The authors carried out a trial on cemented crowns (IPS e.max Press®, Ivoclar Vivadent AG, Liechtenstein), and found a mean fracture force of 901.67 N ± 102.05 for the abutment under study and of 480.01± 174.46 for the control group, that is the zirconium abutments (Procera AllZirkon®, Nobel Biocare, Sweden).

An in vitro study carried out by Mitsias and cols. (12) compared a zirconium abutment (Astra Tech, Y-TZP®, Sweden) with a titanium abutment, both repaired with metallic crown. The titanium abutment showed significantly better behaviour, both at constant speed (1475 N ±625 for Ti and 690 N ± 430 for Zr) and during cycled trial. As regards the reliability of both types of abutments, titanium abutments showed a 100% reliability rate whereas zirconium abutments lost reliability as the load was increased up to 400 N. 

Northdurft and cols. (13) introduce in their study a new parameter, temperature. They performed a thermocycler trial (105 cycles between 5 and 55ºC) of straight and angled zirconium abutments (ZirDesign®, Astra Tech, Sweden) and found a mean fracture force for the former of 233.68 N± 30.68.

## Discussion

The literature so far published on this subject allows us to observe the behaviour of zirconium abutments under loads and pressure resembling daily mastication. We commonly find a wide variability of fracture force values among similar studies. The difficulty to simulate daily mastication resides in the great amount of factors involved in such process which complicate the design of this kind of tests. 

As a result, although the occlusal forces involved in mastication are well documented, there is no agreement on mean values. Leaving aside individual anatomic and physiologic characteristics, maximum occlusal force is reported to be located in the first molar region, with values ranging from 180 to 850 N, decreasing to values between 95 and 250 N in the incisive region (14-16). Considering these data and the fact that, on average, zirconia abutments fracture occurs around 500-540 N, according to the reviewed articles, we could predict a satisfactory aesthetic behaviour of zirconium abutments but we should be cautious with their use in posterior regions. 

As regards fatigue trials, a mean load of 50 N during 2.4x105 cycles is considered to represent a year long of mastication. Such data are supported by the agreement observed between clinical and laboratory studies. In this sense, zirconium abutments still show some shortcomings, but we must bear in mind that the lesser the tension on repaired abutments the better their fatigue behaviour and the more satisfactory the results of in vitro studies on abutments without prosthetic crowns.

**Table 1:**
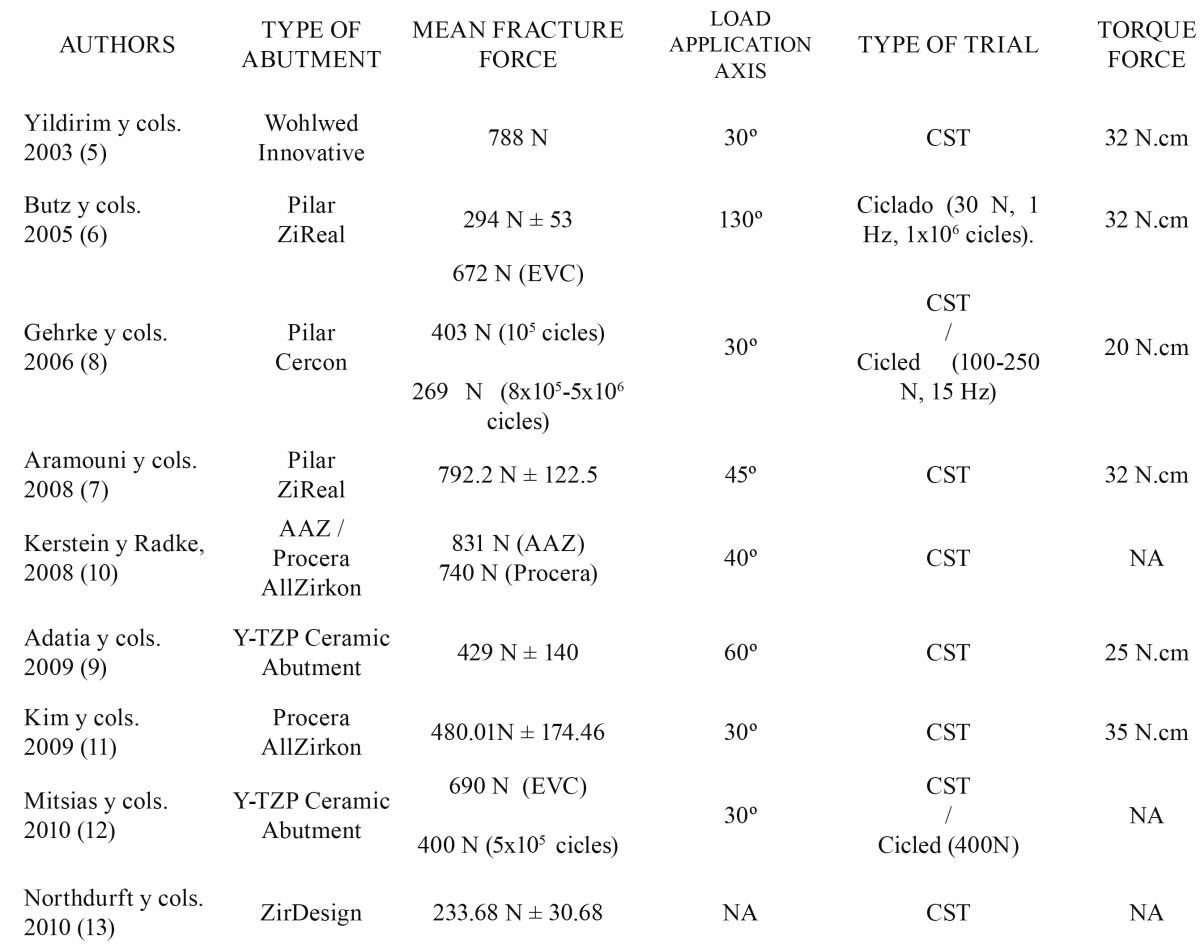
Mechanical trials on zirconium abutments. (NA: not available; CST: constant speed trial).

Current clinical tests show the favourable outcome of zirconium abutments in the incisive region, even in the canine-premolar region, in instances of unitary implant-supported rehabilitations (17-19). Nevertheless, neither our review nor others carried out by different authors, show adequate clinical evidence as regards the use of this type of abutments in the molar region (20).
